# Nonlinear associations of birth weight and gestational age with initial newborn hearing screening referral in a large Chinese neonatal cohort

**DOI:** 10.3389/fpubh.2026.1879289

**Published:** 2026-07-07

**Authors:** Xiaozhe Yang, Yue Li, Cheng Wen, Xiaohua Cheng, Jinge Xie, Yu Ruan, Chuan Wang, Wei Zhang, Qingjia Cui, Yiding Yu, Lin Deng, Shan Gao, Beier Qi, Hui En, Hui Liu, Xinxing Fu, Lihui Huang, Demin Han

**Affiliations:** 1 Key Laboratory of Otolaryngology Head and Neck Surgery (Capital Medical University), Ministry of Education, Beijing, China; 2 Department of Otolaryngology-Head and Neck Surgery, Beijing Tongren Hospital, Capital Medical University, Beijing, China; 3 Beijing Institute of Otolaryngology, Beijing, China; 4 Maternal and Child Health Hospital of Chao Yang District, Beijing, China; 5 Centre for Ear Sciences, Medical School, The University of Western Australia, Crawley, WA, Australia; 6 Ear Science Institute Australia, Subiaco, WA, Australia

**Keywords:** birth weight, concurrent newborn hearing and genetic screening, gestational week, hearing loss, newborns

## Abstract

**Background:**

The potential risk factors for concurrent newborn hearing screening and genetic screening remain unclear, posing challenges to the prevention of hearing loss. Although gestational age and birth weight are known to influence hearing development, their associations with newborn hearing screening referral remain controversial due to limited large-scale validation.

**Methods:**

This retrospective population-based cohort study included 76,460 newborns who underwent concurrent newborn and genetic screening. Restricted cubic splines and piecewise linear models were used to identify threshold effects in the associations of birth weight and gestational age with initial hearing screening referral, second hearing screening outcomes, and genetic screening results. Sensitivity analyses were conducted by including sex and Apgar scores.

**Results:**

We identified non-linear, U-shaped associations between birth weight, gestational age and the odds of referral at initial hearing screening. A birth weight of 3,297 g and gestational age of 39.19 weeks were key change points. Infants above or below these points had increased odds of referral at initial hearing screening. Neither second hearing nor genetic screening results showed significant associations with both factors. Sex-stratified analysis showed male infants had higher odds of referral at initial hearing screening, with similar non-linear patterns observed for gestational age. Adjusting for Apgar scores confirmed these results, with the thresholds at a birth weight of 3,297 g and gestational age of 39.27 weeks.

**Conclusion:**

Birth weight and gestational age were associated with initial hearing screening referral only. The lowest referral odds occurred around 3.3 kg and 39 weeks of gestation, which may help identify newborns who require closer follow-up after initial screening.

## 1 Introduction

Hearing loss (HL) is a significant public health concern. The World Health Organization (WHO) reported that over 5% of the world’s population has disabling HL, including 34 million children (1). Several determinants of hearing capacity, including genetic, biological, psychological, and environmental factors, impact the ears at different stages of life to prevent or induce HL (1). During the perinatal period, the causative factors leading to HL include hypoxia, birth asphyxia, hyperbilirubinemia, low birth weight, and other perinatal morbidities and their management (2–5).

It is reported that very low birth weight (<1,500 g) and preterm birth (<37 weeks) are significant risk factors for a range of adverse neonatal outcomes, including neurodevelopmental impairments, intellectual disabilities, and sensory disorders. However, the specific nature of the relationship between the broader spectrum of birth weight, gestational age, and their associations with newborn hearing screening referral remain less clear and warrants further population-based validation (6–10).

Universal newborn hearing screening (UNHS) has become an important part of global neonatal care aimed at the early detection of HL to prevent delayed or hindered speech and language development. The UNHS program has been in place and developed in China over 30 years, bringing significant socioeconomic effects (11, 12). Simultaneously, advances in genetic screening have enabled the identification of genetic risk factors for congenital HL, further aiding in timely intervention. Concurrent newborn hearing and genetic screening in Beijing has been conducted since 2012 and plays important roles not only in the early detection, diagnosis, and intervention of congenital HL but also in predicting late-onset and progressive hereditary HL (13).

Previous studies on hearing screening or diagnostic outcomes in preterm infants and those with low birth weight have highlighted the increased risk of failing UNHS and the increased likelihood of temporary or permanent HL diagnosis among preterm and low birth weight infants. This risk often occurs due to an underdeveloped auditory system and transient conditions such as middle ear effusion (9, 10, 14–16). However, the potential impact of gestational age and birth weight on auditory development remains to be validated in large populations. Moreover, specific thresholds for optimal screening outcomes across the spectrum of these factors have not been established, nor has their intersection with the results of increasingly common concurrent newborn hearing and genetic screening programs been sufficiently examined in a sizable cohort study.

This large-scale cohort study examined the associations of birth weight and gestational age with initial hearing screening referral, second hearing screening referral, and genetic screening referral. The objectives were to quantify how these perinatal parameters were associated with screening referral patterns and to characterize their potential non-linear relationships. Elucidating these biological determinants is critical for enabling early follow-up after newborn hearing screening. This knowledge forms the cornerstone for developing precise paediatric preventive strategies and promoting lifelong auditory health.

## 2 Methods

### 2.1 Study design and population

This retrospective population-based cohort study analysed retrospective data collected from 24 maternal and childcare centres in Beijing, China. A total of 93,465 newborns were initially eligible, all of whom underwent concurrent newborn hearing and genetic screening. The population enrolment process has been described in a previous publication (11). After excluding 17,005 newborns with missing key covariate or outcome data, 76,460 newborns were included in the final analysis. We collected information on hearing screening techniques, screening results in the UNHS, and audiological results of these cases, as well as concurrent newborn hearing and genetic screening findings. Additionally, we obtained information on the newborns’ sex, birth weight, gestational age, foetal multiplicity, residence, and Apgar scores at 1, 5, and 10 min. The study timeline included birth, initial hearing screening within 72 h after birth, genetic screening 3 to 7 days after birth, second hearing screening by 42 days of age for infants who received a refer result at initial screening. Diagnostic audiological assessment was generally performed for infants with a refer at the second screening; however, diagnostic outcomes, long-term audiological follow-up data, and loss-to-follow-up information were not available for all infants in this retrospective study. We adopted the complete-case analysis for missing data.

### 2.2 Concurrent newborn hearing and genetic screening

Concurrent newborn hearing and genetic screening was defined as the screening approach in which newborns underwent both newborn hearing screening and genetic screening. Hearing screening was performed within the framework of the universal newborn hearing screening program implemented in China. According to the “Technical Specifications for Newborn Hearing Screening (2010 Edition),” for normal neonates, initial hearing screening was performed using transiently evoked otoacoustic emission (TEOAE) testing within 72 h after birth. For infants who received a refer result in the initial screening, a second hearing screening was performed by the age of 42 days. The second screening used the TEOAE testing or TEOAE combined with automated auditory brainstem response (AABR) testing. Newborns in the neonatal intensive care unit (NICU) were screened using AABR before discharge (17). The following were the pertinent TEOAE test parameters: “click” as auditory stimulation with a stimulus intensity of 70–75 dB sound pressure level (SPL). Total reaction intensity ≥10-dB SPL, repetition rate ≥50%, and signal-to-noise ratio (SNR) (at least three frequencies) ≥ 3 dB with background noise <40 dB (A) were the requirements for passing (13, 18, 19). The AABR test parameters were: “click” as auditory stimulation with a normalized hearing level (nHL) stimulus intensity of 35 dB; 93 times per second; 16 kHz sampling rate; up to 15,000 times signal superposition; spectrum ranging from 700/750 Hz to 5,000 Hz; and background noise <45 dB (A) (20). Genetic screening was conducted according to previously described methods (13). All neonates were screened for 15 genetic variants across four important genes using the Deafness Gene Variant Detection Array Kit (CapitalBio): gap junction beta-2 (*GJB2*), solute carrier family 26 member 4 (*SLC26A4*), gap junction protein beta 3 (*GJB3*), and mitochondrial DNA 12S ribosomal RNA (*mtDNA 12S rRNA*) (13, 21). Blood samples were collected from neonates born in Beijing where hearing screening was also performed. The genetic screening outcomes were categorized as wild-type or positive (variants in *GJB2*, *SLC26A4*, *GJB3*, or *mtDNA*). A wild-type result indicated that no variant included in this targeted screening panel was detected but did not exclude other genetic causes of hearing loss.

### 2.3 Covariates

We selected several covariates to account for potential confounding factors that may influence the association between birth weight (weight of the newborn at birth, measured in grams), gestational age (number of weeks of gestation at the time of delivery, calculated from the mother’s last menstrual period), and initial hearing screening referral, second hearing screening referral, and genetic screening referral. The covariates included sex; foetal multiplicity (number of foetuses or singleton or multiple gestations); residence (residential location of the newborn, categorized as Beijing, other cities, or outside China); and Apgar scores at 1, 5, and 10 min after birth (providing a quick assessment of the newborn’s health, with scores ranging from 0 to 10, based on the evaluation of five criteria: heart rate, respiratory effort, muscle tone, reflex irritability, and color). We included these covariates in the statistical models to control for potential confounding effects on the associations between birth weight, gestational age, and screening outcomes.

### 2.4 Statistical analysis

All statistical analyses were performed using SAS, version 9.4 (SAS Institute, Cary, NC, USA) and R, version 4.2.0. *p* < 0.05 indicated statistical significance. We calculated descriptive statistics for all covariates, including sex, foetal multiplicity, residence status, and Apgar scores at 1, 5, and 10 min. Continuous variables, including birth weight, gestational age, and Apgar scores, were summarized as means and standard deviations (SDs). Categorical variables, including initial hearing screening outcomes, second hearing screening outcomes, genetic screening outcomes, sex, preterm birth, foetal multiplicity, and residence, are presented as frequencies and percentages.

We applied the *χ^2^* test for categorical variables and the *t-*test or analysis of variance (*ANOVA*) for continuous variables to assess the differences in covariates according to the outcomes of the initial hearing, second hearing, and genetic screening, respectively. The dependent variables were hearing and genetic screening results, categorized as pass/referral (initial and second hearing screening) or wild type/positive (genetic screening). The independent variables included birth weight, gestational age, sex, and Apgar score.

We performed restricted cubic splines (RCS) analysis using the ‘rms’ package in R (22), with four knots (placed at the 5th, 35th, 65th and 95th percentiles) to flexibly model the association of birth weight, gestational age with the initial hearing screening, the second hearing screening, and genetic screening outcomes. Odds ratios (ORs) with 95% confidence intervals (CIs) were calculated to quantify the strength of the associations of birth weight and gestational age with initial hearing screening, second hearing screening, and genetic screening outcomes. The lowest OR was selected as the reference value.

The spline models were mutually adjusted for birth weight and gestational age. We tested for potential non-linearity using a likelihood ratio test, comparing the model with only a linear term to the model with linear and cubic spline terms. We also applied piecewise two-line models to explore the potential non-linear associations among birth weight, gestational age, screening outcomes, change points, and OR were calculated.

We conducted several sensitivity analyses. First, we conducted sensitivity analyses to evaluate the robustness of our findings, particularly by examining the impact of gestational age and birth weight in subgroups stratified by sex. Additionally, we examined the shapes of the relationships between birth weight, gestational age, initial hearing screening, second hearing screening, and genetic screening outcomes after including Apgar scores.

## 3 Results

### 3.1 General characteristics of study population

This study included 76,460 neonates who underwent concurrent newborn hearing and genetic screening between January 2019 and December 2020. Table 1 shows the characteristics of the neonates included in the study according to the newborn hearing and genetic screening results. In this study, 7.2% (5,503/76,460) of the neonates were born preterm. Most newborns were singletons (96.2%), with 3.8% being part of a multifoetal pregnancy (twin or higher-order multiple). Additionally, 56.9% (43,526/76,460) of the neonates were residents of Beijing, 42.8% (32,710/76,460) were residents of cities outside Beijing, and 0.3% (224/76,460) were residents of cities outside China.

**Table 1:** Characteristics of neonates included in the study by initial hearing screening outcomes, second hearing screening outcomes, and genetic screening results (*N* = 76,460).

Characteristics	Initial hearing screening			Second hearing screening			Genetic screening		
	Pass	Referral	*χ^2^* (*p*-value)	Pass	Referral	*χ^2^* (*p*-value)	Wild type	Positive	*χ^2^* (*p*-value)
Screening Outcomes	73,834 (96.6%)	2,626 (3.4%)		76,037 (99.4%)	423 (0.6%)		72,697 (95.1%)	3,763 (4.9%)	
Gestational age^a^	38.7 (1.6)	38.3 (2.2)	−8.44 (<0.0001)	38.7 (1.7)	38.7 (1.8)	−0.17 (0.866)	38.7 (1.7)	38.7 (1.7)	0.46 (0.643)
Preterm birth^b^			*χ^2^* (*p*-value)			*χ^2^* (*p*-value)			*χ^2^* (*p*-value)
Yes	5,155 (6.7%)	348 (0.5%)	149.26 (<0.0001)	5,475 (7.2%)	28 (0.0%)	0.21 (0.645)	5,239 (6.9%)	264 (0.3%)	0.20 (0.659)
No	68,679 (89.8%)	2,278 (3.0%)		70,562 (92.3%)	395 (0.5%)		67,458 (88.2%)	3,499 (4.6%)	
Birth weight^c^			*t* (*p*-value)			*t* (*p*-value)			*t* (*p*-value)
	3293.1 (484.4)	3221.1 (607.1)	−6.01 (<0.0001)	3290.4 (489.2)	3319.7 (530.7)	1.13 (0.257)	3290.3 (489.2)	3294.1 (494.0)	0.62 (0.536)
Sex			*χ^2^_CMH_* (*p*-value)			*χ^2^_CMH_* (*p*-value)			*χ^2^_CMH_* (*p*-value)
Female	35,676 (46.7%)	1,031 (1.3%)	83.45 (<0.0001)	36,540 (47.8%)	167 (0.2%)	12.41 (0.002)	34,863 (45.6%)	1844 (2.4%)	10.29 (0.006)
Male	38,156 (49.9%)	1,595 (2.1%)		39,495 (51.7%)	256 (0.3%)		37,833 (49.5%)	1918 (2.5%)	
Unspecified	2 (0.0%)	0 (0.0%)		2 (0.0%)	0 (0.0%)		1 (0.0%)	1 (0.0%)	
Foetal multiplicity			*χ^2^_CMH_* (*p*-value)			*χ^2^_CMH_* (*p*-value)			*χ^2^_CMH_* (*p*-value)
Singleton	71,098 (93.0%)	2,468 (3.2%)	37.19 (<0.0001)	73,157 (95.7%)	409 (0.5%)	0.26 (0.608%)	69,947 (91.5%)	3,619 (4.7%)	0.02 (0.891)
Multifoetal pregnancies	2,736 (3.6%)	158 (0.2%)		2,880 (3.8%)	14 (0.0%)		2,750 (3.6%)	144 (0.2%)	
Residence			*χ^2^_CMH_* (*p*-value)			*χ^2^_CMH_* (*p*-value)			*χ^2^_CMH_* (*p*-value)
Beijing	42,062 (55.0%)	1,464 (1.9%)	3.60 (0.165)	43,286 (56.6%)	240 (0.3%)	0.06 (0.973)	41,337 (54.1%)	2,189 (2.9%)	4.63 (0.099)
Other cities	31,552 (41.3%)	1,158 (1.5%)		32,528 (42.5%)	182 (0.2%)		31,142 (40.7%)	1,568 (2.1%)	
Outside China	220 (0.3%)	4 (0.0%)		223 (0.3%)	1 (0.0%)		218 (0.3%)	6 (0.0%)	
Apgar scores			*t* (*p*-value)			*t* (*p*-value)			*t* (*p*-value)
Apgar at 1 min	9.9 (0.6)	9.8 (0.7)	−7.46 (<0.0001)	9.9 (0.6)	9.8 (0.5)	−1.82 (0.069)	9.9 (0.6)	9.9 (0.6)	−1.41 (0.159)
Apgar at 5 min	10.0 (0.4)	9.9 (0.4)	−5.00 (<0.0001)	10.0 (0.4)	10.0 (0.3)	−1.51 (0.133)	10.0 (0.4)	10.0 (0.3)	−1.82 (0.069)
Apgar at 10 min	10.0 (0.2)	10.0 (0.3)	−4.92 (<0.0001)	10.0 (0.2)	10.0 (0.2)	−1.62 (0.105)	10.0 (0.2)	10.0 (0.2)	−0.98 (0.327)

^a^Gestational age (weeks); ^b^Preterm birth is defined as gestational age <37 weeks; ^c^Birth weight is presented in grams.

Data are presented as n (%) or mean (SD). Percentages are calculated using the entire cohort as the denominator. Values <0.05% are displayed as 0.0%. χ^2^_CMH_ values were calculated using the Cochran–Mantel–Haenszel test to account for stratification.

**Figure 1:**
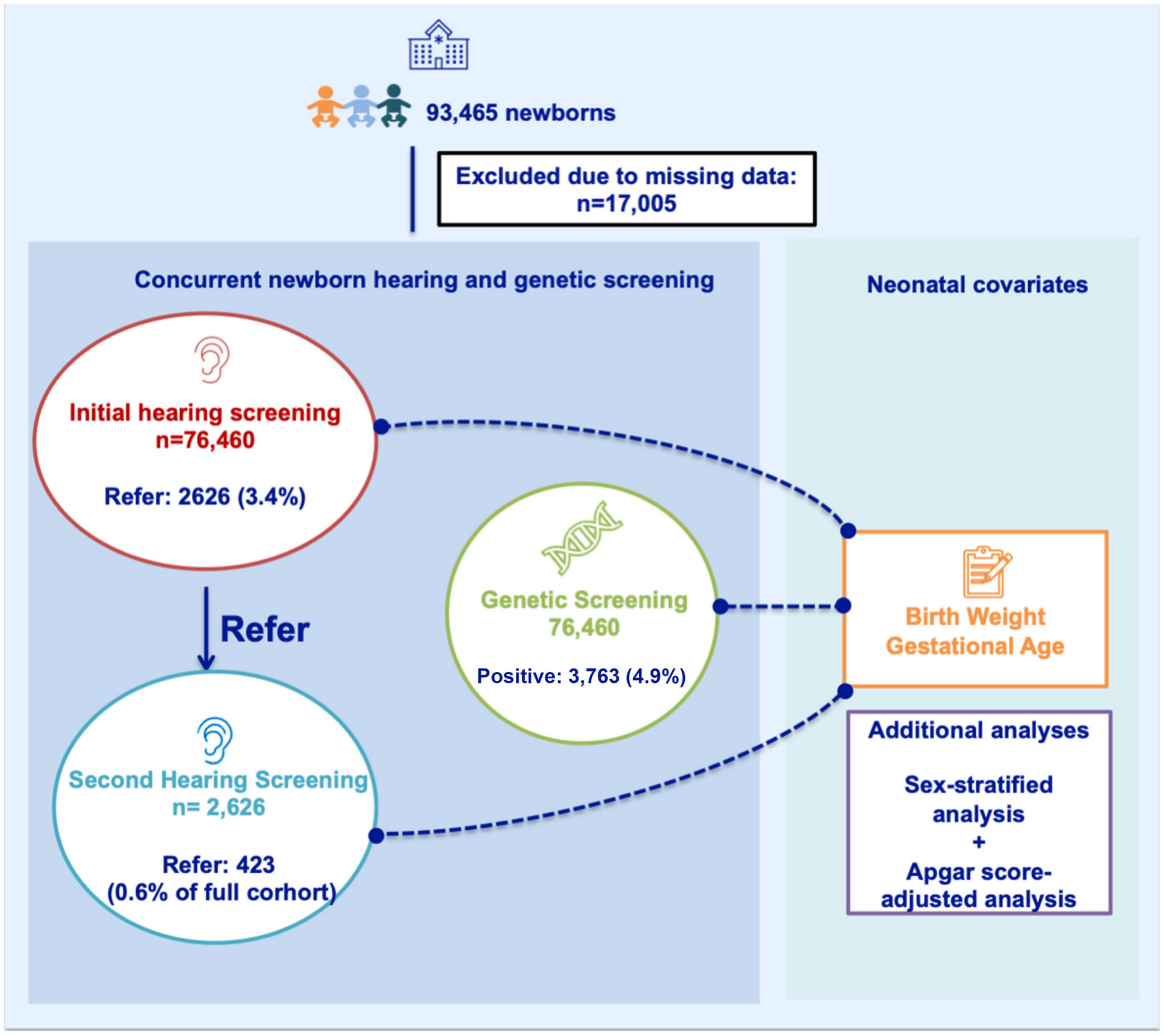
Flow diagram of the study. In the current study, the cohort consisted of 93,465 neonates. After excluding 17,005 newborns with missing key covariate or outcome data, 76,460 newborns were included in the final complete-case analysis. Initial hearing screening was performed for all included newborns, among whom 2,626 (3.4%) received a refer result. Infants with an initial refer result underwent second hearing screening, and 423 newborns were referred for diagnostic audiological assessment. Genetic screening was also performed for all included newborns, and 3,763 (4.9%) were referred.

Of the 76,460 included neonates, 72,337 passed the concurrent newborn hearing and genetic screening, 5.4% (4,123/76,460) received a refer result, and 3.4% (2,626/76,460) were referred in the initial hearing screening. In the second hearing screening, ultimately 0.6% (423/76,460) were referred for an audiological diagnosis. Additionally, 4.9% (3,763/76,460) of the newborns were positive for deafness-associated variants based on genetic screening. Among the referred infants with concurrent newborn hearing and genetic screening, 63 newborns (0.1%) failed the screening (Figure 1).

### 3.2 Associations of birth weight and gestational age with referral at initial hearing screening

**Figure 2:**
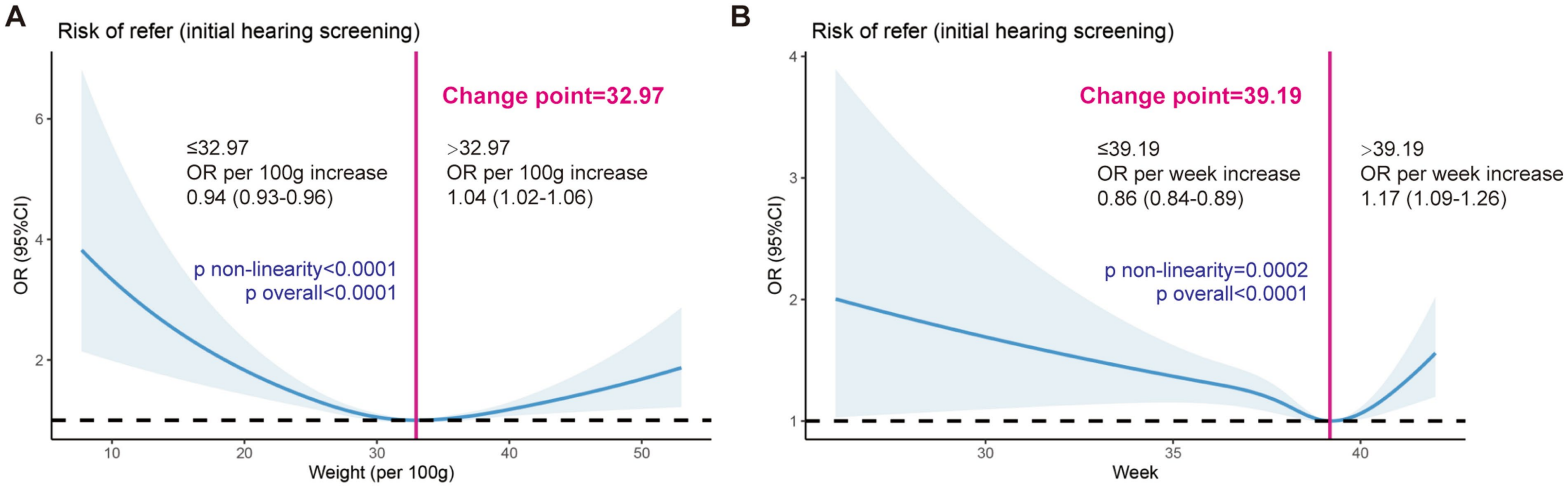
Associations of birth weight or gestational age with initial hearing screening referral. Restricted cubic spline models showing the associations of **(A)** birth weight and **(B)** gestational age with odds of referral at initial hearing screening. Birth weight was modeled in units of 100 g; therefore, the change point of 32.97 corresponds to 3,297 g. The change point for gestational age was 39.19 weeks. The reference value was the point with the lowest estimated odds ratio. Shaded areas represent 95% confidence intervals. Change points and segment-specific odds ratios were estimated using piecewise two-line models. The spline models were mutually adjusted for birth weight and gestational age.

The piecewise two-line model analysis revealed significant associations between birth weight and initial hearing screening referral as well as between gestational age and hearing screening (Table 2). Regarding birth weight, we identified a change point of 3,297 g. Below this change point, each 100-g increase in birth weight was associated with lower odds of referral at initial hearing screening (OR 0.94, 95% CI 0.93–0.96, *p* < 0.0001), whereas above this change point, each 100-g increase was associated with higher odds of referral at initial hearing screening (OR 1.04, 95% CI 1.02–1.06, *p* < 0.0001). This association was pronounced among infants with lower and higher birth weights, as also identified by the piecewise two-line model (Figure 2A).

**Table 2:** Associations between birth weight or gestational age and initial hearing screening referral in neonates^a^.

	Change point	Range	OR per unit increase (95% CI)^b^	Z-value	*p*-value
Birth weight (100 g)	32.97	≤32.97	0.94 (0.93–0.96)	−7.179	<0.0001
		>32.97	1.04 (1.02–1.06)	4.870	<0.0001
Gestational age (weeks)	39.19	≤39.19	0.86 (0.84–0.89)	−9.083	<0.0001
		>39.19	1.17 (1.09–1.26)	4.149	<0.0001

^a^Based on piecewise two-line models, the spline models were mutually adjusted for birth weight and gestational age. ^b^ORs represent the odds of receiving a refer result at initial hearing screening per 100 g or per week increase in the respective variable. OR, odds ratio; CI, confidence interval.

Regarding the association between gestational age and outcomes of initial hearing screening, we identified a change point of 39.19 weeks, with significant effects observed below and above this threshold (Figure 2B). Below this change point, each additional week of gestation was associated with lower odds of referral at initial hearing screening (OR 0.86, 95% CI 0.84–0.89). Conversely, gestational age above this change point was associated with increased odds of referral at initial hearing screening (OR 1.17, 95% CI 1.09–1.26).

### 3.3 Associations of birth weight and gestational age with second hearing and genetic screening outcomes

**Figure 3:**
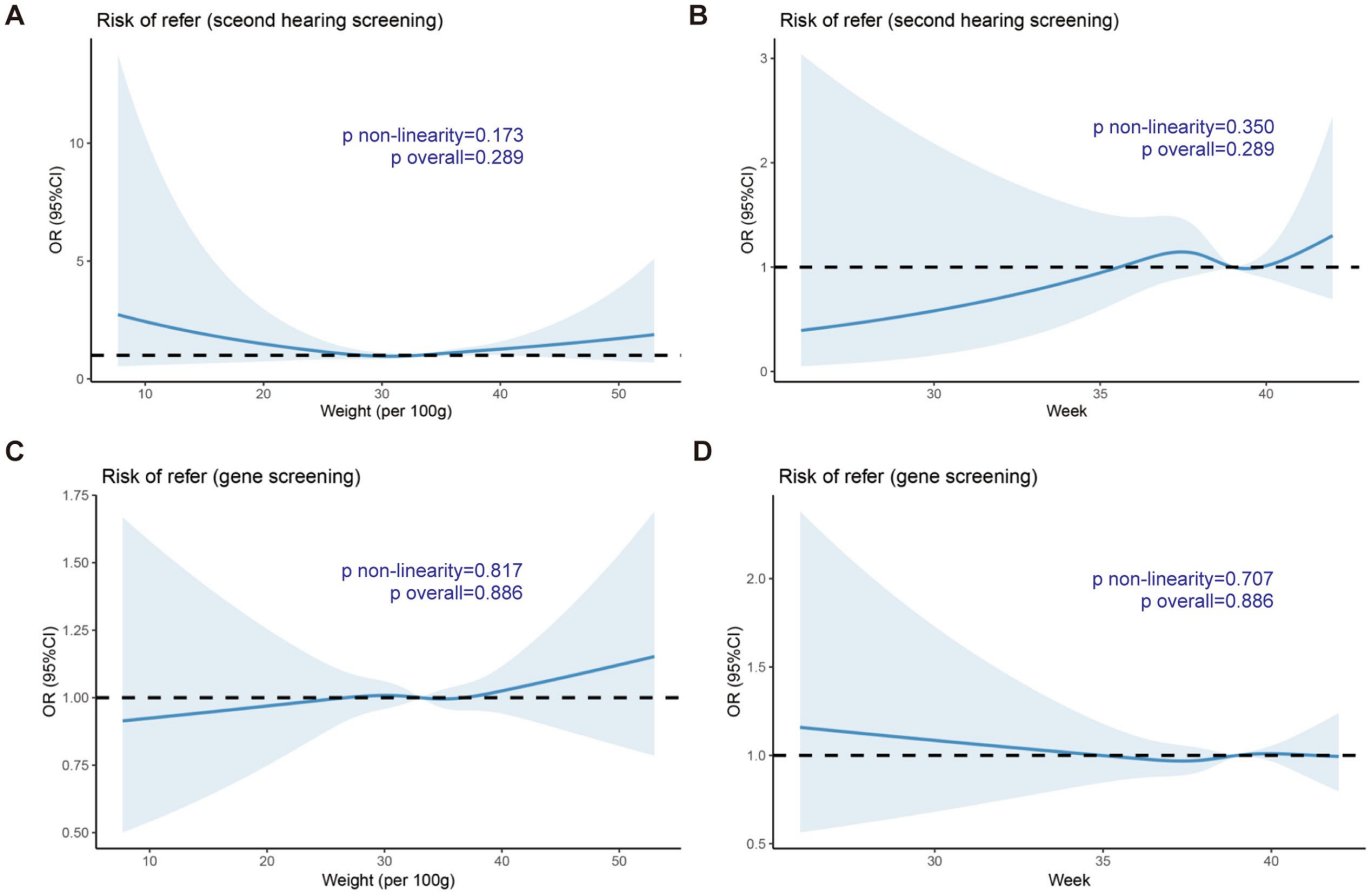
Associations of birth weight or gestational age with the risk of second hearing screening referral and genetic screening referral. Restricted cubic spline models showing the associations of **(A)** birth weight and **(B)** gestational age with odds of referral at second hearing screening, and the associations of **(C)** birth weight and **(D)** gestational age with odds of genetic screening referral. Birth weight was modeled in units of 100 g. The reference value was the point with the lowest estimated odds ratio. Shaded areas represent 95% confidence intervals. The spline models were mutually adjusted for birth weight and gestational age.

Figure 3 presents the associations of birth weight or gestational age with second hearing screening outcomes and genetic screening results. The association between each 100-g increase in birth weight and the odds of referral at second hearing screening was not statistically significant. (*P* for non-linearity = 0.173, *P* overall = 0.289; Figure 3A). The odds of referral at second hearing screening showed a fluctuating trend with each additional week of gestation; however, the association was not statistically significant (*P* for non-linearity = 0.350, *P* overall = 0.289; Figure 3B). Birth weight and gestational age were neither significantly associated with the odds of positive results at genetic screening (Figures 3C,D).

### 3.4 Sex stratification

**Figure 4:**
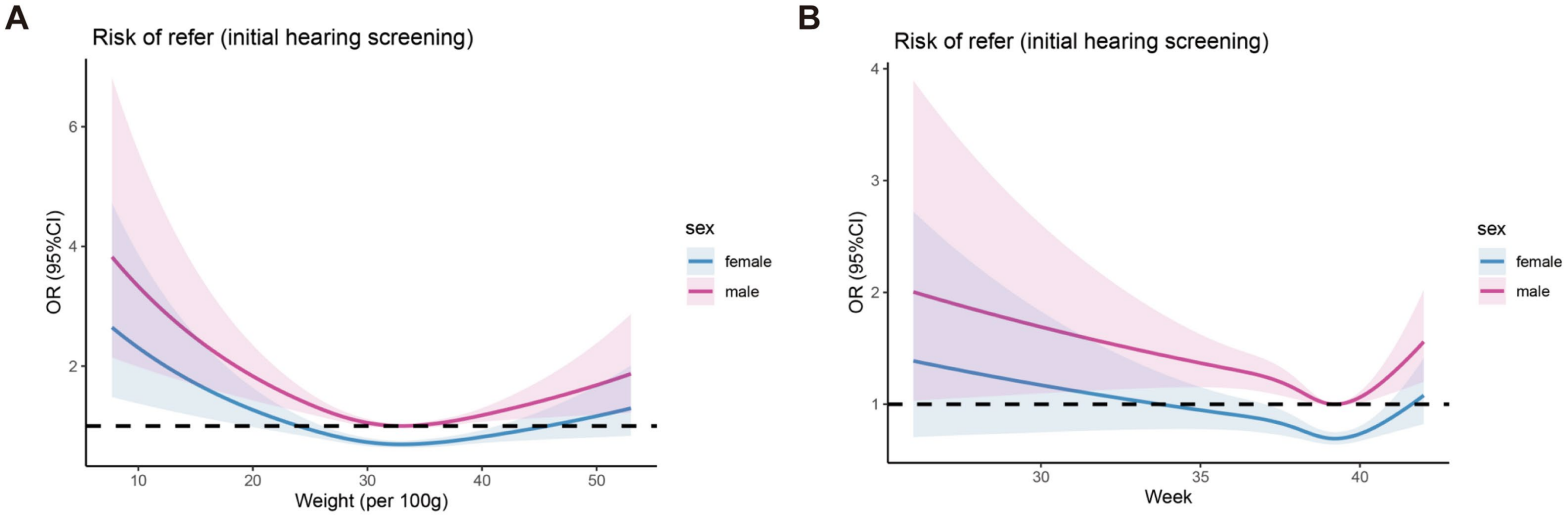
Association of birth weight or gestational age with initial hearing screening referral stratified by sex. Restricted cubic spline models showing the sex-stratified associations of **(A)** birth weight and **(B)** gestational age with odds of referral at initial hearing screening. Birth weight was modeled in units of 100 g. The reference value was the point with the lowest estimated odds ratio. Shaded areas represent 95% confidence intervals. The spline models were mutually adjusted for birth weight and gestational age.

When stratified by sex, we observed significant associations between birth weight, gestational age, and initial hearing screening referrals in both male and female newborns (Figure 4, Table S1). Regarding birth weight, male newborns had higher odds of referral at initial hearing screening than females (reference group) (OR 1.45, 95% CI 1.34–1.57, *p* < 0.0001). Similarly, for gestational age, male newborns had higher odds of referral at initial hearing screening than female newborns (OR 1.44, 95% CI 1.33–1.56, *p* < 0.0001).

### 3.5 Sensitivity analyses

The results of the sensitivity analyses to assess the robustness of the primary findings showed that after adjusting for Apgar scores, the associations between birth weight, gestational age, and outcomes of the initial hearing screening remained mostly consistent with the primary results (Supplementary Figure S1; Supplementary Table S2). Moreover, for each 100-g increase in birth weight or each additional week of gestational age, the odds of referral at the second hearing or genetic screening outcomes were comparable to those from the primary models, with minimal changes in effect size (Supplementary Figure S2). Similarly, the sex-stratified associations of birth weight and gestational age with screening outcomes were similar to those in the primary analysis after adjustment for Apgar scores (Supplementary Table S3; Supplementary Figure S3). The inclusion of the Apgar scores in the sensitivity analysis confirmed the robustness of the primary findings.

## 4 Discussion

### 4.1 Summary of principal findings

In one of the largest newborn cohorts in China, this study identified birth weight and gestational age as factors associated with initial hearing screening referral. The results demonstrated significant associations between these perinatal parameters and the odds of referral at initial hearing screening. The lowest odds of initial hearing screening referral occurred around 3.3 kg and 39 weeks of gestation. Newborns with birth weight or gestational age above or below these estimated points tended to have higher odds of referral at initial hearing screening.

### 4.2 Interpretation of the study and comparison with previous studies

Our study contributes to a broader understanding of perinatal factors that influence neonatal hearing health. Previous studies have established low birth weight and preterm birth as key risk factors for hearing loss; however, few have provided threshold-based insights across the full spectrum of birth weight and gestational age (23–27). Moreover, few large-scale population-based studies have jointly examined hearing and genetic screening outcomes in relation to these perinatal indicators. By assessing both screening methods, our study provides a more comprehensive picture of the impact of birth weight and gestational age on concurrent newborn hearing and genetic screening.

Our findings are consistent with previous evidence that low birth weight and preterm birth may be associated with adverse auditory outcomes. However, in the present study, the significant associations were observed for referral at initial hearing screening rather than confirmed hearing loss. The estimated change point around 3.3 kg was consistent with literature suggesting that infants born with low birth weight are at greater risk of developmental delays, including hearing impairment (5, 7, 28–30). However, we extended this knowledge by showing that the odds of referral at initial hearing referral persists above this birth weight point, albeit at a lower level. This novel finding suggests that the relationship between birth weight and these outcomes is more nuanced than was previously believed. These results are consistent with those of studies in different populations that have demonstrated the increased vulnerability of preterm infants to hearing loss and other developmental issues (8, 10, 24, 25). Notably, the estimated change point occurred around 39 weeks of gestation, further contributing to the understanding of how gestational age influences neonatal health outcomes and emphasizing the importance of gestational maturity in auditory development.

Overall, the relationship between birth weight, gestational age, and the risk of hearing loss remains controversial, with some studies showing mixed results. Meyer et al. observed an increased risk of delayed identification of hearing loss among infants with low birth weight and other socioenvironmental factors, such as public insurance, low maternal education, and rural residency (9). Another study showed that, at the same post-gestational age, newborns with very (28–31 weeks) and extremely (<28 weeks) preterm birth had lower hearing screening pass rates compared with those with higher gestational ages, despite having normal hearing (14). Research from Italy reported that preterm birth and neonatal suffering were key causes of hearing loss (30). Ohl *et al.*, observed that birth weight <500 g and preterm birth before the 34th week of pregnancy did not significantly affect sensorineural hearing loss (23). Another retrospective study reported that low birth weight was associated with the presence of HL during newborn hearing screening and diagnosis (16).

In the present study, birth weight and gestational age were closely associated with the initial hearing screening but not with the second hearing screening and genetic screening outcomes. It suggests that birth weight and gestational age may be associated mainly with early screening referral rather than persistent hearing screening referral or genetic risk. One possible explanation for the lack of association in the second screening could be that the initial screening should be conducted in 48 h after birth, and infants who fail this need to be rescreened within 42 days. The lack of association in later screenings might be attributed to the natural development of the auditory system over time or the effect of compensatory mechanisms in infants who initially failed the hearing test but later passed (17, 31–33). Initial screening is performed shortly after birth and may be influenced by transient neonatal conditions, including vernix obstruction, residual middle-ear fluid, transient conductive dysfunction, or physiological adaptation. These factors may be more common in infants with suboptimal perinatal conditions and may increase the likelihood of an initial “refer” result. Their resolution before the second screening may partly explain why the associations with birth weight and gestational age were attenuated at the second screening stage. Additionally, the genetic screening used in this study was a targeted panel covering 15 selected variants in four common deafness-related genes. Therefore, a wild-type result in this targeted genetic screening panel did not exclude other pathogenic variants or hereditary causes of hearing loss. Also, some genetic factors might become more pronounced in later stages of life, with genes in genetic screening related to congenital, delayed, and progressive hearing loss, which may diminish the influence of perinatal factors such as birth weight and gestational age on screening referral (34, 35). Thus, while birth weight and gestational age are critical in the early screening phase, genetic predispositions may become more relevant as children grow older and undergo additional assessments.

After sex stratification, the associations between birth weight, gestational age, and screening outcomes remained significant for both male and female infants but were more pronounced in males. Male infants below or above the change points had higher odds of referral at initial hearing screening compared with females. Biological differences in foetal development and susceptibility to environmental factors may contribute to this sex-specific vulnerability (23). These findings add to the growing body of evidence on sex-based disparities in neonatal health outcomes, which have been attributed to differences in neonatal development. In addition, screening methods might be related to the outcomes of hearing screening: a previous study reported that male sex increased risks of false-positive results related to OAE tests (36). In Beijing, we mainly used the OAE and AABR tests for hearing screening (31).

### 4.3 Strengths and limitations

A major strength of this study is the large sample size and the inclusion of concurrent newborn hearing and genetic screening data, which enhanced the validity and generalisability of our findings. Moreover, the use of piecewise two-line models enabled us to identify the estimated change points at which the associations changed, providing nuanced insights into the relationships between perinatal factors and the odds of referral at initial hearing screening. Moreover, our analysis extended beyond birth weight and gestational age by including Apgar scores, a key measure of neonatal conditions at birth, and by performing stratified analyses by sex to enhance the interpretability and robustness of the results.

However, this study has several limitations. First, the observational nature of this study limited causal inferences. Although we adjusted for multiple covariates, residual confounding from unmeasured variables such as maternal health behaviors, environmental exposure, and socioeconomic status, parental hearing status, and family history of hearing loss might still exist. Future studies incorporating more comprehensive clinical, familial, and perinatal information are needed to better evaluate these associations. Second, data on NICU admission status, individual screening modality, exact screening timing after birth, and screening center were not completely available for all infants. Therefore, we could not adjust for or stratify by these factors. Because these variables may be associated with both birth weight/gestational age and hearing screening referral, residual confounding and method-related heterogeneity cannot be fully excluded. Future studies with detailed recording of NICU status, screening modality, screening timing, and center-level practice are needed to further validate these findings. Third, this study focused on newborn hearing screening outcomes rather than their diagnostic audiological outcomes. Systematic diagnostic audiological data, including hearing outcomes after diagnostic referral and long-term audiological status at later ages, such as 1, 3, or 6 years, were not available for all infants. Therefore, future studies with standardized diagnostic tests and audiological follow-up as main outcomes are needed to determine whether birth weight and gestational age are associated not only with early screening results but also with delayed-onset hearing loss, or long-term auditory development. Finally, although this was a multicenter study, the population was limited to neonates in Beijing and the identified change points lacked external validation, which may limit the generalisability of the findings.

## 5 Conclusion

In this retrospective population-based cohort study of 76,460 Chinese newborns, birth weight and gestational age showed non-linear associations with initial newborn hearing screening referral, with the lowest referral odds occurring around 3.3 kg and 39 weeks of gestation. Male infants had significantly higher odds of referral than female infants. Neither second hearing screening results nor genetic screening outcomes were significantly associated with birth weight or gestational age. These findings suggest that birth weight and gestational age are associated with initial hearing screening referral and may help identify newborns who require closer follow-up after initial screening within the framework of universal newborn hearing screening.

## Data Availability

The original contributions presented in the study are included in the article/Supplementary material, further inquiries can be directed to the corresponding authors.
