# Supplementary material for: Nonlinear associations of birth weight and gestational age with initial newborn hearing screening referral in a large Chinese neonatal cohort
Source: Front Public Health. 2026 Jul 7;14:1879289. doi: 10.3389/fpubh.2026.1879289 (PMC13385711; doi:10.3389/fpubh.2026.1879289)
Supplement: Supplementary file 1 [file Data_Sheet_1.DOCX]

Supplementary Material

Table of Contents

[Table S1 Associations of birth weight and gestational age with initial hearing screening referral stratified by sex 2](#_Toc194992604)

[Table S2 Change points and associations of birth weight and gestational age with initial hearing screening outcomes, adjusted for Apgar score 3](#_Toc194992605)

[Table S3 Associations of birth weight or gestational age with initial hearing screening outcomes stratified by sex, adjusted for Apgar score 4](#_Toc194992606)

[Figure S1 Associations of birth weight or gestational age with referral risk in initial hearing screening, adjusted for Apgar score 5](#_Toc194992607)

[Figure S2 Associations of birth weight or gestational age with the risk of hearing loss (from the second hearing screening and genetic screening), adjusted for Apgar score 6](#_Toc194992608)

[Figure 3 Associations of birth weight or gestational age with initial hearing screening outcomes stratified by sex and adjusted for Apgar score 7](#_Toc194992609)

**Table S1 Associations of birth weight and gestational age with initial hearing screening referral stratified by sex**

|  | **Sex** | **OR (95% CI)** | **Z-value** | ***P*-value** |
| --- | --- | --- | --- | --- |
| **Birth weight model** | Female | 1 (ref) ^a^ |  |  |
|  | Male | 1.45 (1.34–1.57) | 8.945 | <0.0001 |
| **Gestational age model** | Female | 1 (ref) ^a^ |  |  |
|  | Male | 1.44 (1.33–1.56) | 8.872 | <0.0001 |

^a^ Female was used as the reference category.

OR, odds ratio; CI, confidence interval.

Estimates were derived from piecewise two-line models within sex stratification. The models were mutually adjusted for birth weight and gestational age.

**Table S2 Change points and associations of birth weight and gestational age with initial hearing screening outcomes, adjusted for Apgar score**

|  | **Change point** | **Range** | **OR per unit increase (95% CI)** | **Z-value** | ***P*-value** |
| --- | --- | --- | --- | --- | --- |
| **Birth weight (100 g)** | 32.97 | ≤32.97 | 0.95 (0.93–0.96) | -6.342 | <0.0001 |
|  |  | >32.97 | 1.03 (1.02–1.05) | 4.305 | <0.0001 |
| **Gestational age (weeks)** | 39.27 | ≤39.27 | 0.88 (0.86–0.91) | -7.512 | <0.0001 |
|  |  | >39.27 | 1.17 (1.08–1.26) | 3.711 | <0.0001 |

OR, odds ratio; CI, confidence interval.

Estimates were derived from piecewise two-line models. The models were mutually adjusted for birth weight and gestational age and additionally adjusted for Apgar Scores.

**Table S3: Associations of birth weight or gestational age with initial hearing screening outcomes stratified by sex, adjusted for Apgar score**

|  | **Sex** | **OR (95% CI)** | **Z-value** | ***P*-value** |
| --- | --- | --- | --- | --- |
| **Birth weight model** | Female | 1 (ref) ^a^ |  |  |
|  | Male | 1.45 (1.33–1.57) | 8.887 | <0.0001 |
| **Gestational age model** | Female | 1 (ref) ^a^ |  |  |
|  | Male | 1.44 (1.33–1.56) | 8.829 | <0.0001 |

^a^ Female was used as the reference category.

OR, odds ratio; CI, confidence interval.

Estimates were derived from piecewise two-line models within sex stratification. The models were mutually adjusted for birth weight and gestational age and additionally adjusted for Apgar Scores.

**
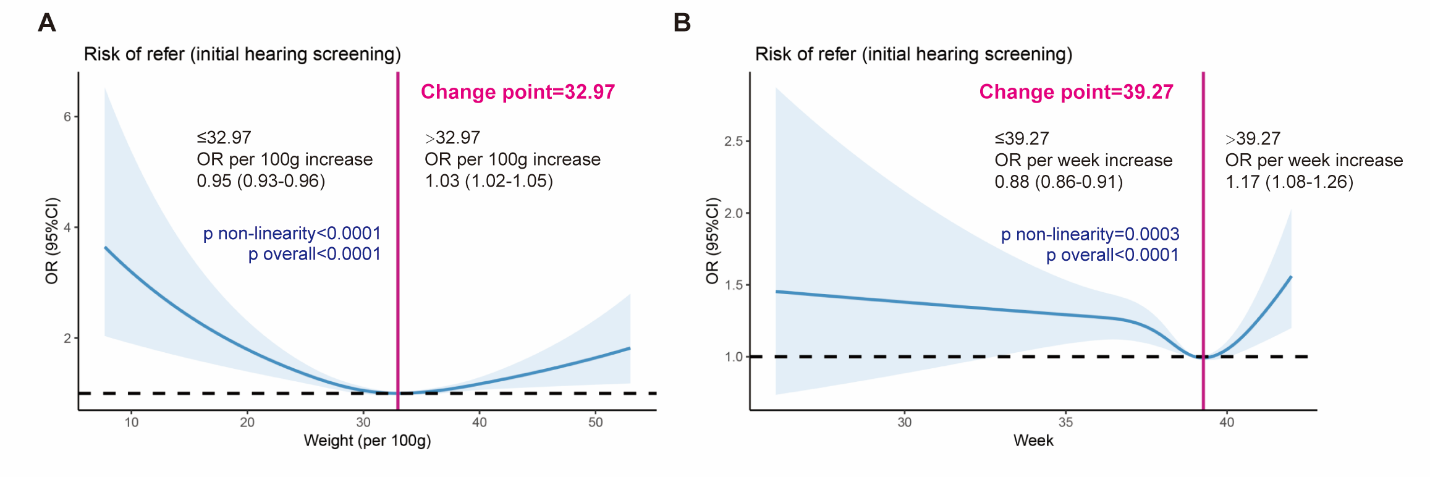
Figure S1. Associations of birth weight or gestational age with referral risk in initial hearing screening, adjusted for Apgar score**

# Restricted cubic spline models showing the associations of (A) birth weight and (B) gestational age with odds of referral at initial hearing screening. Birth weight was modeled in units of 100 g; therefore, the change point of 32.97 corresponds to 3,297 g. The change point for gestational age was 39.27 weeks. The reference value was the point with the lowest estimated odds ratio. Shaded areas represent 95% confidence intervals. Change points and segment-specific odds ratios were estimated using piecewise two-line models. The spline models were mutually adjusted for birth weight and gestational age and additionally adjusted for Apgar Score.

**
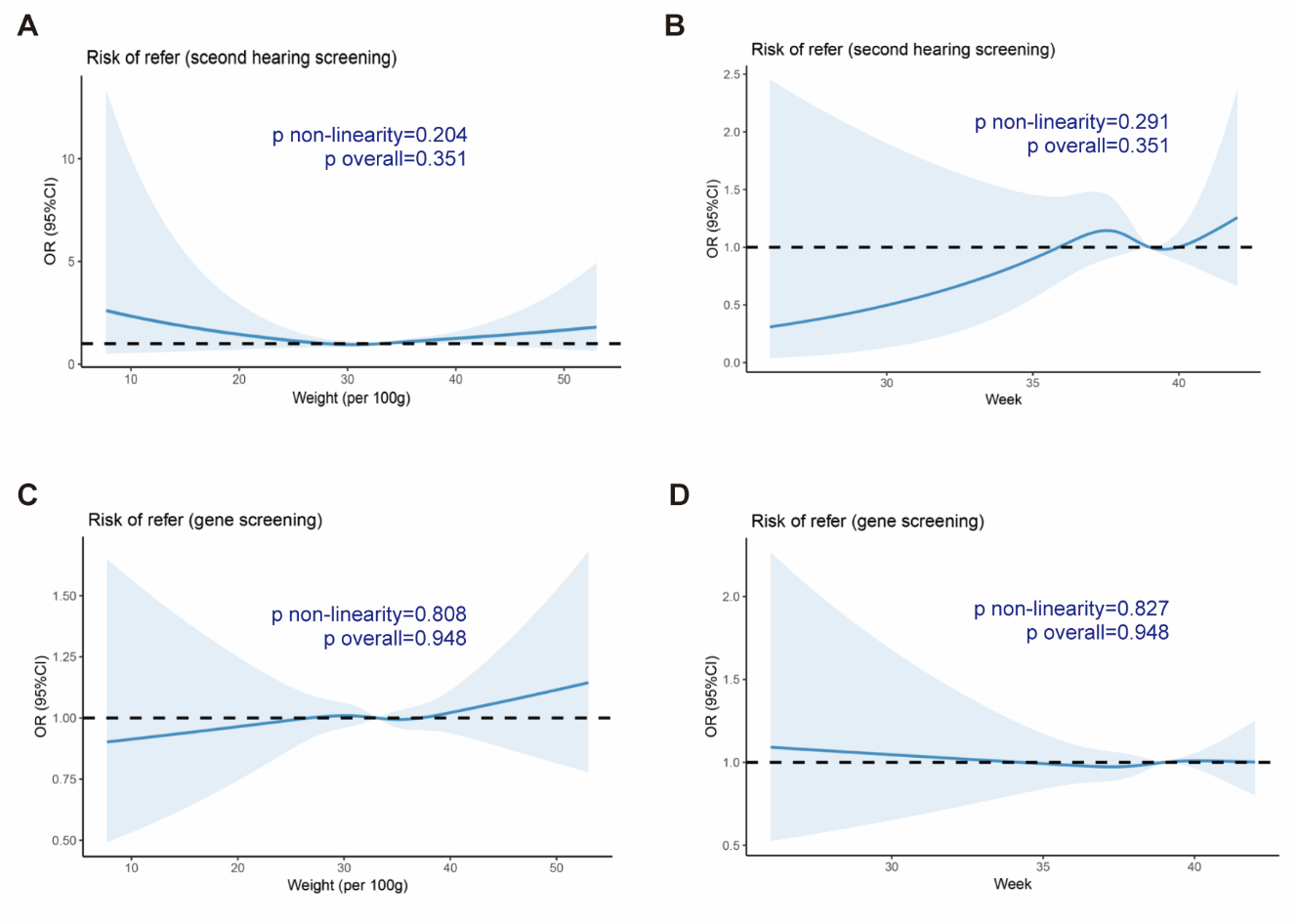
Figure S2 Associations of birth weight or gestational age with the risk of hearing loss (from the second hearing screening and genetic screening), adjusted for Apgar score**

Restricted cubic spline models showing the associations of (A) birth weight and (B) gestational age with odds of referral at second hearing screening, and the associations of (C) birth weight and (D) gestational age with odds of genetic screening referral. Birth weight was modeled in units of 100 g. The reference value was the point with the lowest estimated odds ratio. Shaded areas represent 95% confidence intervals. The spline models were mutually adjusted for birth weight and gestational age, and additionally adjusted for Apgar Score.


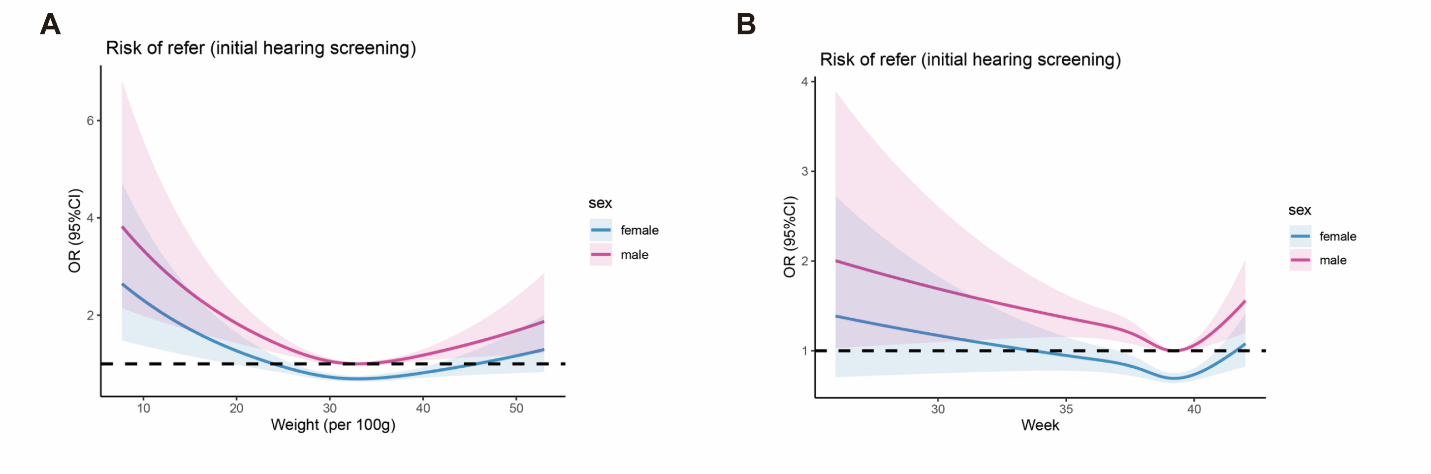
**Figure S3 Associations of birth weight or gestational age with initial hearing screening outcomes stratified by sex and adjusted for Apgar score**

Restricted cubic spline models showing the sex-stratified associations of (A) birth weight and (B) gestational age with odds of referral at initial hearing screening. Birth weight was modeled in units of 100 g. The reference value was the point with the lowest estimated odds ratio. Shaded areas represent 95% confidence intervals. The spline models were mutually adjusted for birth weight and gestational age and additionally adjusted for Apgar Scores.
